# Insects for Food and Feed-Safety Aspects Related to Mycotoxins and Metals

**DOI:** 10.3390/foods8080288

**Published:** 2019-07-26

**Authors:** Pamela Schrögel, Wim Wätjen

**Affiliations:** 1 Postgraduate Course for Toxicology and Environmental Toxicology, Institute for Legal Medicine, University of Leipzig, Johannisallee 28, 04103 Leipzig, Germany; 2 Institute of Agricultural and Nutritional Sciences, Martin-Luther-Universität Halle-Wittenberg, Weinbergweg 22, 06120 Halle/Saale, Germany; 3 Competence Cluster for Nutrition and Cardiovascular Health (nutriCARD), Halle-Jena-Leipzig, 04103 Leipzig, Germany

**Keywords:** mycotoxin, cadmium, arsenic, insect, food, feed, contaminants

## Abstract

Edible insects as an alternative source of protein are discussed as an important contribution to future feed and food security. However, it has to be ensured that the consumption is non-hazardous. This systematic review summarizes findings concerning contaminations of insects with mycotoxins and heavy metal ions (SciFinder, Pubmed, until 26 June 2019). Both kinds of contaminants were reported to reduce growth performance and increase mortality in insects. There was no evidence for accumulation of various mycotoxins analyzed in distinct insect species. However, further research is necessary due to limitation of data. Since the gut content contributes relevantly to the total body burden of contaminants in insects, a starving period before harvesting is recommended. Contrary, accumulation of heavy metal ions occurred to a varying extent dependent on metal type, insect species, and developmental stage. Examples are the accumulation of cadmium (black soldier fly) and arsenic (yellow mealworm). The reported species-specific accumulation and metabolism patterns of contaminants emphasize the importance to assess potential safety hazards in a case-by-case approach. Subject to regular monitoring of contaminants, the general ban in the European Union to use waste in animal feed should also be questioned regarding insect farming.

## 1. Introduction

In the context of an increasing world population, the search for alternative protein sources to support future food security is a highly important topic. According to projections by the United Nations Population Division, a world population of 9.7 billion and 11.2 billion is to be expected by 2050 and by 2100, respectively. This results in an estimated increased demand for worldwide agricultural output by 2050 of almost 50 percent compared to 2013 [1]. Edible insects could contribute to future food and feed security from an economic as well as a sustainable perspective due to their relatively short lifecycle. Furthermore, some of them can transform even waste streams into valuable proteins revealing high feed conversion ratios at low levels of greenhouse gas emission [2,3]. By 2012, approximately 1900 edible insect species were listed worldwide. While in Africa, Latin America and Asia, insects are traditionally implemented in the human dietary, in Western countries entomophagy is still rather uncommon and occasionally associated with feelings of disgust [4]. It is obvious that insect species of interest to be used as animal feed or in human diet in the EU should be non-toxigenic and non-pathogenic towards humans and relevant animals [5]. In Table 1, a selection of insect species with the potential to be used as food or feed is summarized together with information on their life-stage at harvest according to the risk profile related to production and consumption of insects as food and feed by the European Food Safety Authority (EFSA) [6].

Regarding the black soldier fly, for example, larvae, prepupae, and pupae are suitable to be used as feed and food, but not the eggs since ingestion of eggs has been related to a risk to develop intestinal and cutaneous myiasis according to some human case studies [7,8,9,10]. The impact of the composition of the rearing substrates on the development [11] and the nutritional values of the insects [12] as well as their potential as a source of valuable protein in human diets [13] has already been demonstrated. Insects are a rich source of proteins (content ranges from 20 to 76%, depending on the insect species and developmental stage). Furthermore, insects might represent a pool of several micronutrients such as minerals and vitamins [14,15] and additional bioactive compounds with potential beneficial health benefits [16].

An overview of the key regulatory aspects regarding food and feed in the European Union is provided in Table 2.

Insects that are bred by humans and used for the production of food or any other product obtained from animals are considered as livestock in the European Union according to Article 3 (6) of the EC Regulation 1069/2009 [17]. Consequently, the regulations regarding animal feed apply to insect farming as well. While some insect species are capable of converting waste into valuable biomass, under EC Regulation 1069/2009 [17] and EC Regulation 767/2009 [18] it is currently restricted to feed animals on various waste stream materials, such as household waste or manure. In view of an economic industrial-scale process with high conversion rate and high insect protein yields, rearing of insect larvae should be optimized by using a substrate of constant composition and specified quality. Currently, it is also not allowed in the EU to feed processed animal protein to farmed animals, with the exemption of insect oil and hydrolyzed proteins according to EC Regulation 999/2001 [19] on the prevention of transmissible spongiform encephalopathies. Since its amendment EU 2017/893 [20] it has been permitted to use processed insect protein as feed for aquaculture animals. In the feed production, maximum levels are set by the European Commission for contaminants such as heavy metals (e.g., cadmium), pesticides, and mycotoxins (e.g., aflatoxin B1) [21]. For the mycotoxins deoxynivalenol, zearalenone, fumonisins, ochratoxin A and T-2 toxin and HT-2 toxin guidance values are determined by the European Commission [22]. Table 3 gives an overview of the EU maximum levels and guidance values of contaminants in feed. These specifications apply to both the farming of the insects as well as to using insects as animal feedstock.

Veldkamp et al. [23], evaluated the use of insects as an alternative source of protein in pig and poultry feed, similar feasibility studies have been reviewed by Khan [24]. In the context of insects as new feed ingredients, van Raamsdonk et al. [25] presented an overview of legal aspects as well as on feed safety, environmental issues, and efficiency. Several safety issues related to the uses of insects for feed and food was also reviewed by van der Fels-Klerx et al. [26].

The basic principles of EU legislation on contaminants in food are described in Regulation 315/93/EEC [27]: Food containing a contaminant in an amount unacceptable from the public health viewpoint is not to be placed on the market. Furthermore, contaminant levels must be kept as low as reasonably achievable following good practices (ALARA principle) and for specific contaminants, maximum limits must be set to protect public health. EC Regulation 1881/2006 [28] sets maximum levels of certain contaminants such as mycotoxins and metals in specific foodstuffs. With respect to human consumption in the EU, farmed insects and products thereof are covered by the novel food regulation EU 2015/2283 [29]. Under this regulation, food or food ingredients are defined as novel which had not been used for human consumption in the EU before 15 May 1997. Prior to placing on the market in the EU, novel food materials either need to undergo an authorization procedure in accordance with Article 10, which includes a safety assessment regarding human health. On the other hand, traditional food from a third country can be notified in accordance with the notification procedure described in Article 14 and 16 provided a record of safe consumption for at least 25 years. In the special case of farmed insects for food application, it is recommended to consider the potential hazards in terms of microbial hazards, chemical hazards, allergenic potential, environmental hazards, and impact of processing and storage as identified in the EFSA opinion [29,30]. Currently, no insect or insect-based product has been authorized as novel food so far in the EU.

Our review article supports future feed and food safety assessments related to the use of insects as a novel protein source in animal feed and for human consumption by providing a comprehensive summary of current literature data available on potential hazards. In this review, a special focus is laid on two of the major environmental contaminants: Mycotoxins and heavy metals. Besides the potential risk for the health of animals and humans, the impact of both mycotoxins and heavy metals on the insects will also be considered in terms of an economic and safe farming procedure.

## 2. Mycotoxins

**Figure 1:**
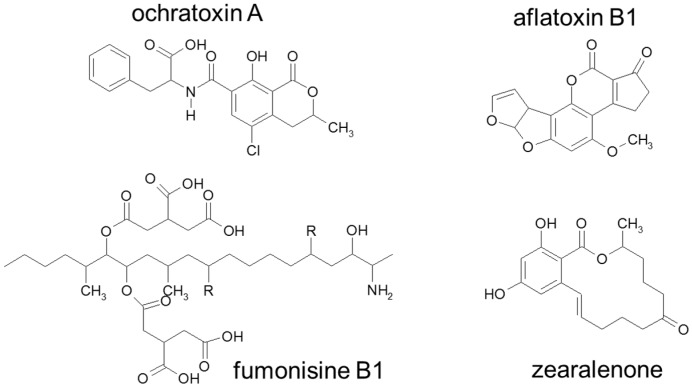
Representative structures of different mycotoxins.

Mycotoxins are low molecular weight secondary metabolites produced by fungi (representative structures of selected mycotoxins are shown in Figure 1) which are capable of causing adverse effects, e.g., in humans. Due to their considerable thermal resistance at typical food preparation temperatures, most mycotoxins might not be inactivated during cooking. Mycotoxins which are most frequently encountered in feed and food materials are briefly presented in the following. The mycotoxin zearalenone (ZEN) is a known endocrine disruptor with estrogenic activity. It is produced by several *Fusarium* species. As several metabolites reveal higher estrogenic activity than zearalenone the European Food Safety Authority defined relative potency factors of ZEN and its modified forms. A relative potency factor of 60 was determined for the most potent metabolite α-zearalenol (α-ZEL) and minor factors have been defined for, e.g., α-zearalanol (α-ZAL) and β-zearalanol (β-ZAL). A group tolerable daily intake (TDI) of 0.25 µg/kg b.w. expressed as ZEN equivalents for zearalenone and its modified forms was set in 2016 by EFSA based on estrogenicity in pigs [30]. Zearalenone can be most commonly found in maize and frequently in wheat in relevant concentrations as evident from analyzed samples throughout Europe reaching concentrations of up to 3170 µg/kg ZEN in maize and up to 424 µg/kg ZEN in wheat [31,32,33,34]. Another class of mycotoxins are fumonisins, secondary metabolites mainly produced by *Fusarium verticilloides* and *Fusarium prolieratum.* These compounds are long chain aminopolyols with two tricarballylic acid side chains. Of most relevance among them, are the B-type fumonisins FB1-FB4 which can be differentiated by the number and position of the hydroxyl-groups in the backbone. A TDI of 1.0 µg/kg b.w. for fumonisin B1 (FB1) was derived by EFSA in 2018 based on an increased incidence of megalocytic hepatocytes found in a chronic study with mice. Considering the limited data on toxicity and mode of action and the structural similarities of FB2-6, it was decided to include FB2, FB3, and FB4 in a group TDI with FB1 [35].

Ochratoxin A (OTA) is a secondary metabolite produced by *Aspergillus* and *Penicillium* species with especially nephrotoxic properties but also neurotoxic, carcinogenic, immunotoxic, and genotoxic activity. It occurs in various food components, such as cereals, nuts, grapes, beer, and coffee. A tolerable weekly intake (TWI) of 120 ng/kg b.w. was derived for OTA by EFSA [36]. Another important class of mycotoxins are the haematotoxic and immunotoxic T-2 toxin (T2) and HT-2 toxin (HT2) which are produced by many *Fusarium* species infesting crop plants. Based on an in vivo subchronic toxicity study in rats, a TDI for T2 and HT2 of 0.02 µg/kg b.w. was established by EFSA [37]. In food products with European origin up to 266 µg/kg T-2 toxin could be detected in oat-based products [38]. The mycotoxin deoxynivalenol (DON) is a secondary metabolite of *Fusarium graminearum*, *Fusarium psedograminearum,* and *Fusarium culmorum* and belongs to the group B of trichothecenes [39]. It co-occurs with the acetylated forms 3-acetyldeoxynivalenol (3-ADON) and 15-acetyldeoxynivalenol (15-ADON) and was frequently detected in almost the half of a total of >26,000 samples of food, feed and unprocessed grains from Europe, with the highest levels found in maize, wheat and oat grains and products thereof. Long term exposure through dietary may lead to reduced weight gain, anorexia, and nutritional efficiency. The TDI of 1 µg/kg b.w. set by the Scientific Committee for Food (SCF) in 2002, was extended to the group of DON and its acetylated derivatives by the Joint FAO/WHO Expert Committee on Food Addivitves (JECFA) in 2010. As deoxynivalenol, also known as vomitoxin, causes gastrointestinal disorders, an acute reference dose (ARfD) was derived at 8 µg/kg b.w. [39]. Aflatoxins are produced by *Aspergillus flavus* and *Aspergillus pararasiticus* and are most commonly found in nuts, figs dried fruits, spices, cocoa beans, crude vegetable oils and maize especially in a warm and humid climate. Due to their distinct genotoxic and carcinogenic potential, naturally occurring aflatoxins were categorized as carcinogenic to humans (group 1) by the International Agency for Research on Cancer (IARC). Hence, no TDI could be derived but regulations at levels considered as low as reasonably achievable were introduced in 1998 by the European Union. Aflatoxin B1 (AfB1) represents the most toxic aflatoxin produced by Aspergillus species and is of great agricultural importance as it occurs most frequently [40].

### 2.1. Effects of Mycotoxins on Insects

Mycotoxins might play a role in terms of economic and safe farming procedure. The effects of mycotoxins on insects have already been addressed by several research groups 50 years ago. For example, spores of *Aspergillus flavus* have been observed to be highly pathogenic to freshly emerged house flies (*Musca domestica* (Diptera: Muscidae)) by ingestion or even by contact [41]. *Aspergillus flavus* showed developmental toxicity also to rice moth larvae [42]. According to the authors, the decrease in growth was related to the toxin rather than to the fungal infection since toxicity did not decrease after sterilization of the infested diet. Feeding aflatoxin to larvae of mosquitos and house flies [43] as well as to young *Heliothis virescence* (Lepidoptera: Nuctuidae) [44] led to high mortality rates. Gudauskas could show that the larval sensitivity to aflatoxin decreased with larval age. One of the first growth studies using larvae of the yellow mealworm (*Tenebrio molitor* (Coleoptera: Tenebrionidae)) to investigate the toxicological impact of feed contaminated with different fungi was published by Reiss [45]: Larvae were reared on bread contaminated with *Aspergillus niger*, *Aspergillus flavus*, *Penicillium expansum*, *Cladosprium herbarum* and *Neurospora sitophila* of which only the latter proved to be non-toxic. In contrast, *A. flavus* most significantly inhibited larval development. As yellow mealworm larvae (*T. molitor*) were also sensitive to mycotoxins produced by *Fusarium* species and by *Myrothecium* species [46] the larvae were supposed to be suitable to screen for those isolates which formed in the field and under storage conditions and should also be suitable to determine the toxicity of fungal metabolites [47]. It also was demonstrated that the growth rate of yellow mealworm larvae is decreased by T-2 toxin, aflatoxin B1, ochratoxin A, and rubratoxin B [48,49]. As a response of confused flour beetles (*Tribolium confusum* (Coleoptera: Tenebrionidae)) to the mycotoxins zearalenone and T-2 toxin Wright et al. found increased fecundity in adults induced by T-2 toxin [50].

Corn earworm larvae (*Heliothis zea* (Lepidoptera: Nuctuidae)) and fall army worm larvae (*Spodoptera frugiperda* (Lepidoptera: Nuctuidae)) were sensitive to trichothecenes as evident in form of reduced growth caused by deoxynivalenol at a concentration of 25 mg/kg. Besides the impact on larval development, the influence of deoxynivalenol and T-2 toxin on the activity of representative midgut detoxifying enzymes in the larvae were analyzed. O-demethylation was induced by a factor of 1.6 in *H. zea* and 6.1 in *S. frugiperda*, respectively, after 48 h rearing on 250 ppm deoxynivalenol. While glutathione transferase conjugation was induced in *H. zea* by exposure to deoxynivalenol at 25 ppm and 250 ppm and to T-2 toxin at 25 ppm, it was inhibited by 10–20% in *S. frugiperda*. Hydrolysis was unaffected in *H. zea* but induced by 30% in *S. frugiperda* by all trichothecenes [51].

Physiological effects as growth performance and respiratory metabolism of yellow mealworm larvae was assessed by Abado-Becognee et al. [52] after exposure to fumonisin B1 (450 µg/g feed). Reduced growth was observed only after consumption of fumonisin B1 for several weeks, as well as a reduced rate of CO_2_ production, reduced feed consumption, and reduced protein metabolism, whereas mortality was not affected. According to the study, approximately 40% of the ingested fumonisin B1 was excreted with feces. This excretion percentage of fumonisin B1 was defined as the difference between the amount taken up by the larvae and the amount detected by titration in the diet at the end of the experiment in relation to the initial concentration in the diet. However, the fumonisin B1 intake could also only roughly be estimated. Thus, the excretion percentage may not be fully reliable. To test the hypothesis that insects may sense and actively avoid fungal colonized grain, Guo et al. [53] observed the parameters of food choice, weight gain and mortality for yellow mealworm larvae (*T. molitor*) when reared on wheat grains colonized by various *Fusarium* species. Compared to the control grain several grains were avoided by the larvae while others were preferred by the larvae, which correlated with larval feeding behavior and weight gain. However, larvae were not always able to sense threats derived from infested grain, as among the preferred grains one grain which was colonized by *Fusarium culmorum* led to increased mortality.

### 2.2. Studies with Controlled Mycotoxin Spiked Substrates

Consumption of mycotoxin-contaminated edible insects may pose a risk to humans or animals. Thus, the effect and fate of mycotoxins ingested by insect larvae need to be explored more in detail. In Table 4, an overview of existing studies conducted under controlled feeding conditions is given with respect to the larvae species, rearing substrate, exposure time, analytes, and treatment prior to analysis.

In one of the first studies with the focus to investigate whether insect larvae can retain mycotoxins in their body when being reared on mycotoxin contaminated substrate, van Broekhoven et al. [54] described a feeding trial with larvae of the species *Alphitobius diaperinus* (Coleoptera: Tenebrionidae), *T. molitor* and *Zophobas atratus* (Coleoptera: Tenebrionidae). The authors selected three commonly encountered mycotoxins zearalenone, ochratoxin A, and T2-toxin for the feeding study. Each of the three mycotoxins was spiked in diets at a relatively high concentration of 500 µg/kg in order to be able to detect mycotoxin contents above the limit of detection by LC-MS/MS (limit of detection: 2 µg/kg for zearalenone and T-2 toxin and 1 µg/kg for ochratoxin A). Depending on their species-specific larval developmental time, the larvae were exposed to the contaminated feed for different periods.

At the end of the feeding period, no significant detrimental effects on larval growth and survival were observed in both larvae fed on control diets and mycotoxin contaminated diets. One population of each larvae species was analyzed for the internal mycotoxin concentration directly after harvesting while the other population was transferred to an uncontaminated control feed for 24 h, 48 h, and 72 h before harvesting and analysis. A third population had been kept fasting for 24 h, 48 h, and 72 h before harvesting and analysis. The toxin uptake by larvae seems to be species-dependent and was low in general: Compared to the high concentration of 500 mg/kg in the feed, only low concentrations were detected in the larvae: The highest values were found in larvae of *T. molitor* with 42 µg/kg dry weight ZEN and 45 µg/kg dry weight T-2 toxin directly after harvesting. Concentrations decreased quickly below the limit of detection when larvae fasted or consumed control feed. In the case of *T. molitor* the concentrations of ochratoxin A decreased faster on fasting compared to feeding on control feed. The authors expected the mycotoxins to be excreted by the larvae through feces causing the rapid decrease in ZEN. Similarly, Bily et al. [62] found constantly decreasing concentrations in larvae of *Helicoverpa zeae* (Lepidoptera: Noctuidae) and *Ostrinia nubilalis* (Lepidoptera: Crambidae) over time, with no ZEN detected in higher developmental stages of pupae and adult. Thus, the recommendation of the authors for an industrial insect rearing would be to follow a fasting period of at least 24 h prior to harvest. The results of the study showed that the tested insect larvae did not retain mycotoxins in their non-metabolized forms. However, no investigation on ingestion, excretion, and possible metabolism of mycotoxins by edible insects have been included in this study.

The same research group analyzed the presence of the mycotoxin deoxynivalenol (DON) in larvae of *T. molitor* and in larval feces [55]. Larvae were grown on wheat flour naturally contaminated with, among other mycotoxins, DON at a concentration of 4.9 mg/kg and the modified forms deoxynivalnol-3-glucoside (DON-3G) and 15-acetyl-DON (15-ADON) at concentrations of 300 µg/kg and 86 µg/kg, respectively. In another feeding group, wheat flour artificially spiked with DON was used. Regarding larval growth and survival rate, no significant difference was observed between the DON containing diets and the control diet. No DON and no acetylated derivatives (3-ADON and 15-ADON) were detected in larvae of any of the feeding groups, regardless of being analyzed directly after harvest or being analyzed after 24 h of fasting.

This study included also the assessment of the excretion of the larvae. The excretion level of DON through larval feces in the case of larvae grown on artificially DON-spiked feed (41%) was nearly 3-times the level of larvae grown on naturally contaminated wheat flour (14%). A potential interpretation of the substantial remaining fraction of DON that could not be detected was, according to the authors, that *T. molitor* might be able to metabolize deoxynivalenol and sequester or excrete these metabolites. The reason for the great difference in excretion levels of DON after feeding on naturally contaminated wheat flour compared to artificially spiked wheat flour could not be clarified. The authors speculated that either the presence of other fungal metabolites in the naturally contaminated flour might impair the excretion of DON. Alternatively, these mycotoxins could be of less stability or matrix effects might lead to a change in the bioavailability of DON. Sanabria et al. [56,57] reported a feeding trial using larvae of *T. molitor* grown on grain naturally contaminated with DON. The grain was sorted into different levels of DON contamination from 0.2 up to 12 mg/kg. After 30 days of exposure to the contaminated feed, the larvae were analyzed for effects on survivability and mycotoxin concentration. The survival rate tended to be higher in the highest DON-diet compared to all other feeding groups. Such an effect was not observed by Broekhoven et al. [55]. Contrary to the findings of van Broekhoven et al. [55], DON was measurable in all insect larvae even after 24 h fasting prior to analysis. Compared to the concentration in the feed, the levels of DON found in the insect larvae did not significantly differ between the feeding groups and were low in general ranging from 97 to 190 µg/kg. However, only small fractions below 6.5% of the presumably ingested DON were detectable in the larval bodies in all feeding groups decreasing from 6.3 to 1.1% with increasing mycotoxin content in the diet. In the residue, 15 to 6% of ingested DON was found unmetabolized, and the metabolite 3-acetyl-DON (3-ADON) was only partially present in the residue samples. Despite the huge fraction of undetected mycotoxin, the authors conclude that larvae of *T. molitor* apparently accumulate DON only at low levels. A major difference in the experimental setup between this study and the study of van Broekhoven [56] was the much longer feeding period, which was nearly doubled, and the age of the larvae (seventh to ninth instar vs. five weeks old larvae). Larvae of 7th to 9th instar are considered to be between 60–80 days old. Purschke et al. [58] investigated the impact of substrate contamination with mycotoxins, heavy metals, and pesticides on the growth performance and composition of larvae of the black soldier fly (*Hermetia illucens* (Diptera: Stratiomyidae)) intended for the use in the feed and food chain.

Regarding the exposure to mycotoxins, the larvae were reared on a substrate of ground corn grains naturally contaminated different mycotoxins (e.g., 4600 ± 1400 µg/kg DON, 860 ± 260 µg/kg ZEN, 88 µg/kg aflatoxin B1, 17 µg/kg aflatoxin B2, 46 µg/kg aflatoxin G2, and 260 µg/kg ochratoxin A). After 10 days the insect larvae were removed from the residual substrate by washing prior to weighing and analysis. No negative impact was observed on the survival rate and the generation of larval biomass compared to the control. None of the mycotoxins present in the feed was detectable in the larvae, despite the lack of a fasting period prior to analysis. However, in the residual material, mycotoxins could be detected. The concentrations of mycotoxins were comparable to those in the spiked feed, except for the concentration of DON which was significantly higher in the residual substrate than in the feed. As a possible explanation, the authors discussed that DON could have been present in a masked form in the feed which remained undetected by conventional analytics leading to an underestimation of the initial concentration of DON in the feed. In masked form, DON may be bound to a carbohydrate or protein matrix as catalyzed by plant enzymes within the detoxification process [63]. Insects might be able to reconvert the masked form into free DON during digestion potentially explaining the increased DON concentration observed in the residual substrate. As shown by Berthiller et al. [64], lactic bacteria commonly present in the intestinal flora can hydrolyze deoxynivalenol-3-glucoside (DON-3G) back to the free DON form. Based on the finding that mycotoxins did not accumulate in the larvae, the authors brought up the potential future perspective to use larvae of *H. illucens* to convert feed substrate which exceeds the allowed levels of mycotoxins in animal feed into valuable insect biomass for feed or non-food application. In this study, however, no recovery assessment on how much of the incorporated mycotoxins could be detected and no investigation of possible metabolites of the mycotoxins was included.

Bosch et al. [59] investigated the tolerance and accumulation of aflatoxin B1 by larvae of the black soldier fly (*H. illucens*) and the yellow mealworm (*T. molitor*). As rearing substrate poultry feed spiked with aflatoxin B1 (AfB1) in nominal concentrations ranging from 0.01 to 0.5 mg/kg dry feed was used. The exposure period was 10 days for the black soldier fly larvae and ended in case of yellow mealworm larvae as soon as the first pupa was observed. Larvae of the yellow mealworm were analyzed directly after harvest while larvae of the black soldier fly were reared on non-contaminated feed for two days prior to analysis. AfB1 in the feed did not affect the survival rate and the bodyweight of the larvae which indicates a high tolerance to AfB1 for both species up to the highest measured dose of 0.415 µg/kg. Besides the analysis of the parent mycotoxin in this study, the analysis was extended to the possible AfB1 metabolite aflatoxin M1 (AfM1). Consistently with the results from Purschke et al. [58], larvae of the black soldier fly (*H. illucens*) did not accumulate aflatoxin B1 as levels of AfB1 and AfM1 were below the limit of detection. In the yellow mealworm larvae, AfB1 could be detected in increasing concentrations of 0.16 to 1.44 µg/kg with increasing AfB1 content in the feed while AfM1 concentrations were also below the limit of detection. To separate the amount of AfB1 in the gut of the yellow mealworm larvae from the whole insect body, in an additional trial, yellow mealworm larvae were also reared on uncontaminated feed for two days prior to analysis. For the highest dose group, the AfB1 concentrations in larvae dropped significantly by a factor of 3.5, comparing 1.4 µg/kg analyzed directly after harvest with only 0.41 µg/kg after two days on uncontaminated feed. This proves that the gut content considerably contributes to the mycotoxin body burden in the insect larvae. In this study, attention was also paid to the fact that high amounts of AfB1 remained undetectable for both insect species as evident from mass balance calculations. For black soldier fly larvae, the amounts of AfB1 lost were in the range of 83 to 95.1% of the amount provided in the feed with similar values of 89 to 95.6% for yellow mealworm larvae. One notable finding was that AfM1 could be detected in the residual substrate of yellow mealworm larvae amounting to 0.9 to 1.7% of the AfB1 provided by feed. Evidence of AfM1 suggests at least a partial metabolism of AfB1 into AfM1 by yellow mealworm larvae. The fact that no AfM1 was found in the case of black soldier fly larvae may indicate species-specific differences in metabolic pathways. The elucidation of the poor recovery of AfB1, however, would require an elaborate investigation of other possible degradation pathways of AfB1. In the feeding study of Camenzuli et al. [60], the potential accumulation of the mycotoxins AfB1, DON, OTA, and ZEN in larvae of the lesser mealworm (*A. diaperinus*) and of the black soldier fly (*H. illucens*) was investigated using commercial wheat-based rearing substrate spiked with the mycotoxins. Both insect species are considered well suited for rearing insects on industry-scale for the use as feed and for human consumption. The concentrations of each mycotoxin were chosen to be 1, 10, and 25 times the maximum EC limits or guidance values for each mycotoxin for complete animal feed. Mixtures of all four mycotoxins have been included with an average of 8- to 20-fold increase of the EC limits or guidance values. According to the different developmental time, different exposure periods were realized for each insect species. After the feeding period, the larvae were placed on non-contaminated feed for two days prior to analysis.

Consistently with results published previously [54,55,59], larvae of both insect species seemed to tolerate high levels of mycotoxins, either as a single contaminant or as a mixture of all four, as no significant differences in survival rate and growth rate were observed compared to the control group. In black soldier fly larvae, DON, ZEN, and OTA could be quantified even in concentrations several orders of magnitude below the concentrations in the feed. In contrast, in lesser mealworm larvae (*A. diaperinus*) all mycotoxins present in the feed were below the limit of quantification. In all residual materials of black soldier fly larvae amounts of mycotoxins could be detected in concentrations slightly above the concentrations in the feed, except for AfB1, which was slightly below the concentration in the feed. In the case of lesser mealworm larvae, the mycotoxin concentrations in the residual material were comparable to the concentration in the feed. In addition to the mycotoxins AfB1, DON, OTA, and ZEN, selected metabolites were analyzed in the larvae and in the residual substrate. In black soldier fly and lesser mealworm larvae, all analyzed AfB1 metabolites aflatoxicol and AfM1 and all analyzed DON metabolites 3-ADON, 15-ADON and DON-3-glucoside were present below the limit of quantification. Only the ZEN metabolites α-zearalenol and β-zearalenol were present in quantifiable but low amounts in black soldier fly larvae. Quantifiable metabolites in the residual material of both larvae were aflatoxicol, α-zearalenol and β-zearalenol, the latter two were found in 40 to 50 times higher concentrations in black soldier fly larvae than in lesser mealworm larvae. In the residual material of lesser mealworm larvae also AfM1 was quantifiable while all DON metabolites were below the limit of quantification in both cases. Mass balance calculations based on the concentration of the mycotoxins in the spiked feed in relation to the quantified amounts of mycotoxins and their metabolites in the larvae, and the two residual materials, originating during the period of rearing on contaminated feed and originating during the subsequent two days of rearing on uncontaminated feed have been performed: Despite taking into account the additional metabolites aflatoxicol, aflatoxin P1 and aflatoxin Q1 compared to considering only AfM1 as in the study of Bosch et al. [59], the mass balance for AfB1 in black soldier fly larvae resulted in only less than 20% of the parent compound. This result confirms the high fractions of undetected AfB1 found by Bosch et al. [59]. For the lesser mealworm (*A. diaperinus*) larvae, a much higher mass balance of AfB1 was determined between 56% and 80% which indicates only limited metabolism of AfB1 by these larvae. With respect to DON, between 55–80% could be accounted for in trials with the black soldier fly and 80–96% in the trials with the lesser mealworm when fed on a mixture of mycotoxins pointing to a different metabolism pattern between both insect species. The majority of OTA and ZEN was excreted in the non-metabolized form by the lesser mealworm larvae as evident from the high mass balance of more than 88%. In contrast, substantial amounts of the ZEN metabolites α-zearalenol and β-zearalenol were formed by black soldier fly larvae, both together accounted for more than 50% of the mass balance. Comparing the trials analyzing mycotoxins with trials investigating a mixture of all four mycotoxins, this study indicated that the metabolisms of insect larvae is not influenced by the presence of other mycotoxins. This is an important finding as usually in naturally contaminated feed, a co-culture of several mycotoxins is found. Regarding OTA, over 97% could be accounted for in case of lesser mealworm larvae, suggesting that almost no metabolism takes place. The comparably low mass balance of 50% for OTA in the case of black soldier fly, however, could not be clarified. This may be related to the formation of other metabolites which have not been considered in this study. Apart from the “loss” of mycotoxins, the authors conclude that larvae of both insect species did not accumulate mycotoxins in their body. Even when reared on substrates exceeding the EC limits and guidance values by factor 25, the insect larvae were compliant with EC limits and guidance values of mycotoxins for animal feed.

Recently, Niermans et al. [61] investigated the biological impact and the metabolic conversion of the mycotoxin zearalenone (ZEN) in yellow mealworm (*T. molitor*) larvae. Different from other studies they conducted a short-term experiment of four weeks and a long-term experiment of eight weeks. The larvae were exposed to ZEN in three different scenarios: Rearing substrate was wheat flour either spiked with pure ZEN or blended with wheat flour which was artificially contaminated by incubation with *Fusarium graminearum*, both diets at levels of 500 and 2000 µg/kg, respectively. In the third scenario, wheat flour blended with naturally contaminated wheat flour at levels of approximately 600 µg/kg and 900 µg/kg was used. The chosen doses resemble results of ZEN values found in samples of wheat and maize in Europe [31,32,33]. Prior to analysis larvae were kept fasting for 24 h. No changes in mortality were observed for any of the feeding group suggesting a high tolerance to ZEN. However, after eight weeks of exposure larvae reared on naturally contaminated feed gained significantly more weight than larvae reared on the other substrates, increasing by 37% and 62% for the low and high dose group compared to the control. This result is in accordance with findings from van Broekhoven et al. [55], which demonstrated enhanced weight gain of *T. molitor* larvae when fed on substrates naturally contaminated with DON compared to artificially spiked DON-substrates or uncontaminated control.

**Figure 2:**
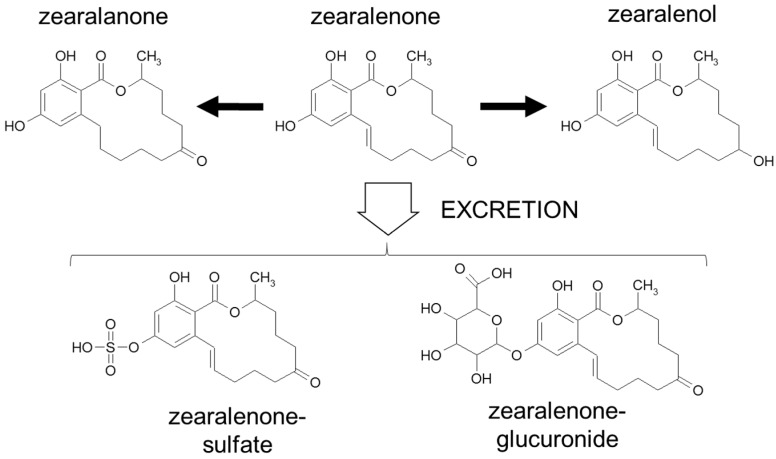
Metabolic pathways of zearalenone (general overview). Shown are only the C-14-linked glucuronides and sulfates of zearalenone, C-16-conjugates may also be formed and are known from in vitro studies [61,65].

Besides ZEN, several known phases I and phase II metabolites were considered in this study, yet, as standards were not available for all metabolites, the analysis was not quantitative. A very general overview of possible metabolic pathways of ZEN is given in Figure 2.

Neither after short-term nor long-term exposure, ZEN or ZEN metabolites were present in detectable amounts in the larvae, while ZEN could be detected in all residue samples. The metabolites α-zearalenol and β-zearalenol were also detected in all residue samples even in the case of rearing substrates that were free of α-zearalenol and β-zearalenol. In some feeding groups, more than 50% of the presumably consumed ZEN remained undetected in the larvae, in unconsumed feed and in larval excretion which confirms findings from Bosch et al. [59] and Van Broekhoven et al. [54]. The reductive metabolites α-zearalenol and β-zearalenol accounted for a fairly high proportion of up to 30% of the total ZEN intake. This metabolic pathway was also observed by Camenzuli et al. [60] for larvae of the black soldier fly. Phase II ZEN metabolites ZEN-14-sulfate (ZEN14Sulf) and in some cases zearalenol-sulfate (ZELSulf) were detectable in the naturally contaminated feed as well as in feed containing artificially contaminated wheat flour while not in the spiked feed or the control feed. No phase II metabolites were detected in the larvae of all feeding groups, but ZEN14Sulf and ZELSulf were found in the residues of the groups reared on naturally and artificially contaminated feed. The fact that no phase II metabolites were found in residues of the feeding group with pure ZEN-spiked substrate suggests that no sulfation occurs in the larvae of *T. molitor*. However, by comparing the HPLC-signals of the naturally contaminated feed with the signals of the resulting residue sample it became evident that ZEN14Sulf—which was a dominant signal in the feed—was reduced in the residual material with two additional evolving signals for the reductive ZELSulf metabolites. This suggested that both free ZEN and ZEN14Sulf were converted either by the larvae or due to contact of unconsumed feed with intestinal bacteria originating from larval feces. A major finding of the study was that no ZEN or ZEN metabolites accumulate in the larvae. Furthermore, it was concluded, that no ZEN-related substances were detectable in the larvae after 24 h of fasting. Thus, the authors concluded regarding feed and food safety that the majority of the ingested toxin was excreted fast and efficiently and should be negligible after a fasting period of 24 h. However, the considerable formation of the metabolite α-zearalenol as potent estrogen might impair the reproduction capability of *T. molitor* in the long term.

### 2.3. Metabolism of Mycotoxins

In literature, consistently low levels of mycotoxins could be found in the larvae after ingestion. Literature data suggest that the metabolic activity of the insect larvae is influenced as a response to the ingested mycotoxins. In 1990, Dowd et al. [51] showed the induction of detoxifying enzymes after exposure to trichothecenes (in vitro metabolism experiments). In experiments using midgut enzymes isolated from larvae of *H. zeae* (in vitro), AfB1 could only be metabolized to aflatoxin P1 (AfP1) and an O-demethylated product of AfB1 after larvae had consumed feed containing plant toxins as xanthotoxin, coumarin or indol-3-carbinol, that are associated to co-occur with AfB1 in plants infested with *Aspergillus* species. Moreover, their results indicated induction of CYP 450 enzymes, such as CYP 321A1 by those plant toxins. As supported by molecular modeling CYP 321A1 was able to form the metabolite AfP1 [66]. The induction of metabolic activity by phytochemicals as well as synthetic substances in *H. zeae* was also confirmed by Wen et al. [67].

In insect larvae with high tolerance to AfB1 such as the yellow mealworm efficient conversion of AfB1 to the metabolites aflatoxicol and in minor amounts aflatoxin B2a (AfB2a) as well as aflatoxin M1 (AfM1) was evident from in vitro experiments, while no epoxidation product could be detected [68]. However, Zeng et al. demonstrated by co-incubation of the CYP inhibitor piperonyl butoxide (PBO) with the AfB1-contaminated diet that the pupation rate could be significantly recovered. This result indicates that the toxicity of AfB1 to larvae of *H. zeae* is mediated by the same metabolic bioactivation mechanism as known for vertebrates. Analogous bioactivation was proven in larvae of caterpillar *Tichoplusioa ni* (Lepidoptera: Noctuidae) [69]. Contrary to these results, bioassays with honeybees (fed with AfB1 and OTA in the presence/absence of PBO) provided evidence that inhibition of CYP 450 enzymes results in higher toxicity of AfB1 [70]. To investigate the capability to convert AfB1, Niu et al. [71] conducted further in vitro metabolism studies with midgut enzymes of larvae of the navel orangeworm (*Amyelois transitellla* (Leptidoptera: Pyralidae)) which reveal a relatively high AfB1 tolerance. Two polar metabolites (not further characterized) were identified by HPLC analysis and assumed to result from hydroxylation as a detoxification pathway of AfB1. Besides various reports on phase I metabolism, De Zutter [72] succeeded in detecting the phase II metabolite DON-3-glucoside (DON-3G) for the first time in aphids, which could only be found in plants before.

### 2.4. Conclusion on Accumulation Potential of Mycotoxins

According to the available literature, mycotoxins adversely affected survival in insects at higher concentrations and reduced growth performance at lower concentrations. However, no accumulation of various mycotoxins was observed in spiked feeding trials with several insect species, even up to a mycotoxin concentration in the rearing substrate of 25 times the maximum EC limits and guidance values, respectively [60]. Based on the reported results, rearing substrates that comply with the current EU limits for AfB1 and guidance values for the mycotoxins DON, ZEN, FB1 and FB2, and OTA should be assumed suitable for insect farming. As it could be demonstrated that the gut content has a great impact on the insect body burden of mycotoxins [59], a starving period of at least 24 h prior to the harvesting of the insects is strongly advised and should become common practice in the context of industrial rearing procedure. However, a substantial portion of mycotoxin ingested could not be recovered in the larvae or the residues. First results suggested a species-specific metabolism pattern [59,60]. As this loss of mycotoxins could not clearly be accounted for, further in vitro and in vivo studies of potential metabolism pathways in insects need to be conducted to gain more insight.

## 3. Heavy Metals and Arsenic

Metals are found naturally ubiquitously in the earth but may become concentrated as a result of human-caused activities. Toxic heavy metals are metals or metalloids that are noted for its potential toxicity. Examples of toxic metals and metalloids are, e.g., cadmium, mercury, lead, arsenic, chromium, cobalt, nickel, and copper. Their toxicity is due to interference with vital cellular components, e.g., replacement of essential metal ions in proteins or induction of oxidative stress. It has to be mentioned that in small quantities, some heavy metals are essential for human health. Decontamination for toxic metals is different from organic toxins since toxic metals cannot be metabolized and have to be excreted from the body. Alternatively, they might be stored as inactive forms (e.g., cadmium bound to metallothionein) and therefore accumulate in the body. Due to this potential accumulation of heavy metals in the food chain, the analysis of these contaminants in insects for food and feed is relevant. Here, we describe the characteristics of some important heavy metal ions.

Mercury occurs in elemental state mercury (Hg^0^), as inorganic mercury (Hg^+^ and Hg^2+^) as well as in organic form, most frequently as methylmercury. Among the targets of inorganic mercury being liver, nervous system, immune system, reproductive, and developmental system, the critical target regarding the tolerable weekly intake (TWI) of 4 µg/kg b.w. as established by EFSA in 2012 was the kidney [73]. After oral intake, methylmercury is absorbed much faster and to a much higher extent than inorganic mercury. Methylmercury is associated with immunotoxic effects and effects on body weight gain, locomotor function, and auditory function. For methylmercury a TWI of 1.3 µg/kg b.w. expressed as mercury, was established [73]. However, in 2015, EFSA recommended limiting the consumption of fish species with high methylmercury such as swordfish, pike, tuna, and hake, in order to minimize the risk of methylmercury to humans most effectively [74]. Cadmium is a nephrotoxic metal and may cause lung damage by chronic inhalation. Due to its estrogenic activity, it may also be considered as an endocrine disruptor. In 2009, EFSA’s CONTAM panel established a tolerable weekly intake (TWI) of 2.5 µg/kg b.w., which was considered still appropriate after a reassessment in 2011 based on the assessment by the Joint FAO/WHO Expert Committee on Food Additives (JECFA) in 2010 [75]. For lead, the CONTAM Panel identified developmental neurotoxicity in young children and cardiovascular effects and nephrotoxicity in adults as the most critical effects regarding the risk assessment of lead in food. As no evidence of a threshold for the lead-induced effects exist, the former provisional tolerable weekly intake (PTWI) of 25 µg/kg b.w. was considered as no longer appropriate [76]. Arsenic occurs in inorganic as well as an organic form, of which the inorganic form is of higher toxicity. Among the inorganic forms, As (III) is considered more toxic than As (V). As arsenic and inorganic arsenic compounds are classified as carcinogenic to humans (group 1) by the International Agency for Research on Cancer (IARC), currently, no maximum levels are established for arsenic in food in the EU [77]. Chromium occurs in different oxidation states. Chromium (III) is an essential nutrient and involved in several physiological metabolic processes. In 2014, a tolerable daily intake (TDI) for chromium (III) of 0.3 mg/kg b.w. was derived by EFSA. Conversely, since chromium (VI) is able to cause cancer, no threshold level for chromium (VI) was established by the Panel on Contaminants in Food Chain (CONTAM Panel) [78]. While short-term exposure to nickel may cause allergic reactions by skin contact but also by ingestion, long-term exposure in animal studies indicated potential reproductive and developmental effects. In 2015, a tolerable daily intake (TDI) of 2.8 mg/kg b.w. was derived [79].

### 3.1. Accumulation of Heavy Metals and Arsenic in Insects

As insects play an important role as part of the food chain in the ecosystem a lot of research work has already been published on their accumulation behavior of metals of which only a selection will be mentioned below. The bioaccumulation of cadmium, manganese, mercury, and zinc via transfer from the aquatic medium to aquatic plants and subsequently to herbivorous insect larvae of *Neochetina eichhorniae* (Coleoptera: Brachyceridae) feeding on the leaves of aquatic plants was investigated by Jamil and Hussain [80]. After one week feeding on leaves which were exposed to different metals before, they detected high levels of cadmium and zinc (compared to lower levels of manganese and mercury) in the insect larvae. Zhang et al. [81] showed the bioaccumulation of cadmium as well as of lead in three of four herbivorous insects. This was analyzed along the food chain from heavy metal contaminated soil to plant, from plant to herbivores insects, and from herbivorous insects to carnivorous insects. In contrast to the study of Jamil and Hussain [80], they additionally detected bioaccumulation of mercury. The extent of bioaccumulation, however, varied depending on the metal investigated as well as the insect species analyzed. Among the metals analyzed, cadmium, lead, and zinc proved to be the most mobile ones and were readily transferred through the food chain consisting of Indian mustard (*Brassica juncea* (Coleoptera: Brachyceridae)), the mustard aphid (*Lipaphis erysimi* (Hemiptera: Aphididae)) and a predatory beetle (*Coccinella septempunctata* (Coleoptera: Coccinellidae)) [82]. Accumulation of cadmium and lead was also observed in earthworm (*Lybrodrilus violaceous*), housefly (*M. domestica*) and dragonfly (*Libellula Luctosa* (Odonata: Libellulidae)) harvested near dumpsite area compared to the same species collected from non-dumpsites [83]. Rearing houseflies from eggs until pupation on substrates contaminated with copper, zinc, lead, and cadmium led to the accumulation of the heavy metals in the body of adult flies. Moreover, larval development, metamorphosis, and survival of the larvae and pupae were adversely affected by the heavy metals tested, except copper. It was shown that houseflies could eliminate cadmium and lead efficiently by bounding the metals to the surface of their exoskeleton which is removed during the molt process [84].

Rosabal et al. [85] observed that the content of metals in the larvae of the phantom midge (*Chaoborus punctipennis* (Diptera: Chaoboridae)) increased significantly after placing them in a lake contaminated with cadmium and selenium. After 16 days of exposure, equal metal levels were reached as measured in larvae of the same species which were indigenous in the contaminated lake. Quantification of the metals in subcellular fractions of the larvae revealed that 60% of cadmium was sequestered by the larvae in a detoxified fraction containing metal-binding proteins and 20% was in sensitive fractions where it could cause toxicity. In contrast, 40% of selenium was apportioned to a metabolically active sensitive fraction. Crawford et al. [86] investigated the accumulation and excretion of dietary copper and cadmium in grasshopper larvae of *Locusta migratoria* R&F (Orthoptera: Acrididae). Larvae were fed on maize contaminated with cadmium at 63 µg/g and maize contaminated with copper at 94 µg/g for between 5 and 20 days and analyzed directly after harvest. Metal analysis suggested that larvae were able to effectively regulate excess of copper in the feed, however, they are not capable of regulating excess of cadmium as evident by an accumulation of cadmium in the larval body proportional to the exposure time to the cadmium-contaminated feed. The metal uptake from natural field soils and from metal spiked soils by larvae of the yellow mealworm (*T. molitor*) was described by Vijver et al. [87]. They observed different metal uptake patterns with almost constant body concentrations of copper and zinc in the larvae independent of the external metal concentration the larvae were exposed to. Concentrations of cadmium and lead, however, were steadily increasing with increasing external metal concentrations. These findings were in accordance to results by Maryanski et al. [88] who observed active regulation of zinc, but the accumulation of cadmium in *Poecilus cupreus* (Coleoptera: Carabidae) beetles after exposure to contaminated feed throughout their entire lifetime. Likewise, crickets showed more efficient regulation of zinc than of cadmium when present in their dietary, suggesting that also crickets tend to accumulate cadmium [89]. It could also be shown that larvae of the beet armyworms developed better cadmium tolerance in terms of survival when they originate from a strain that has been pre-exposed to cadmium throughout ten generations before compared to larvae from an unexposed control strain. The higher cadmium tolerance after multigenerational exposure was assumed to be related to the observed higher antioxidant defense as evident from significantly higher enzymatic activity of catalase in both the gut and the fat tissue of the body [90].

### 3.2. Studies with Controlled Metal Spiked Substrates

Consumption of metal contaminated edible insects may pose a risk to humans or animals. Thus, the effect and fate of different metals ingested by insect larvae need to be explored in detail. In Table 5, an overview of existing studies conducted under controlled feeding conditions is given with respect to the larvae species, rearing substrate, exposure time, analytes, and treatment prior to analysis.

Lindquist [91] examined if cadmium concentrations change during metamorphosis in the black soldier fly. By tracing radio labeled cadmium upon ingestion of a single dose of ^109^Cd, it was shown that molting lowered the cadmium content, but to a lower extent than compared with metamorphosis. At the pupation an almost entire loss of ^109^Cd took place. In a second experiment, continuous intake of different concentrations of cadmium via feed led to a linear rise of cadmium concentration in the larvae and in the adults with increasing cadmium concentration in the feed. This suggests that larvae and adults of *T. molitor* were not capable of regulating cadmium similar to former findings with other species [80,85,87,88]. In newly hatched adults, the cadmium content dropped to 76% of that of the last instar larvae, which was attributed to the molting process [96]. According to the authors, ingested cadmium was distributed to two different pools: One pool was associated with the gut epithelium cells supported by the result published earlier that cadmium was reported to accumulate mainly in the alimentary canal in silkworms assumedly bound to inducible high molecular weight cadmium-binding proteins [97,98,99]. During metamorphosis, the midgut epithelium is renewed and thus, can be excreted via feces. In a later study, Pedersen et al. [100] confirmed the deposition of cadmium in the gut content also for the *T. molitor* with the involvement of cadmium-binding protein of the low cysteine type. The second pool for cadmium was assumed accessible if cadmium penetrated the gut epithelium into other tissues. Thus, this fraction could not be lost during molting and metamorphosis.

In recent time, larvae of the black soldier fly have also attracted attention as being capable to transfer organic waste into valuable protein. However, such substrates might be contaminated with heavy metals which potentially accumulate in the food chain. With a focus on the application as animal feed, Diener et al. [92] were raising the questions if these heavy metals influence the life cycle when present in the feed and to what extent heavy metals accumulate in the pupae of black soldier fly. Chicken feed spiked with cadmium at concentrations up to 50 µg/g, lead at concentrations up to 125 µg/g and zinc at concentrations up to 2000 µg/g was provided to larvae throughout their entire lifetime. Metal analysis was conducted directly after harvest without any fasting period. Neither prepupal weight nor developmental time and sex ratio were affected by any of the three tested heavy metals. Significantly higher concentrations of the heavy metals could be found in prepupae compared to adults. As a possible explanation, it was assumed that larvae defecate before pupation or shortly after adult emergence. The bioaccumulation factors, defined as the metal concentration in the larval body divided by the initial metal concentration in the feed, decreased in the case of zinc with increasing zinc doses in the feed. The authors attribute this result to the active regulation of intracellular zinc uptake in accordance with previous reports [91,101]. For lead, bioaccumulation factors <1 were determined as lead concentrations in the larvae remained below its concentration in the feed. As an explanation, the authors suggested that lead tends to accumulate in the larval exuviae after being stored in granular, metal-containing structures of the cells as described by Hare [102]. In line with literature data [88,96,103], larvae and prepupae were found to accumulate cadmium with bioaccumulation factors >1. Cellular cadmium uptake was assumed to take place through Ca^2+^ channels enabled by the very similar ionic radii of Cd^2+^ and Ca^2+^, according to Braekman et al. [104], who showed in their in vitro experiment that cadmium intake was decreased by the Ca^2+^ antagonist verapamil. Moreover, Braekman et al. [104] observed induction of a protein of the HSP70 family. As potential defense reaction, this protein might contribute to the high tolerance against elevated cadmium concentrations. Regarding the use of the prepupae to produce animal food the authors were not concerned in terms of lead and zinc. However, the accumulation of cadmium could become a critical limitation.

Van der Fels-Klerx et al. [93] compared the uptake characteristics of cadmium, lead, and arsenic between larvae of yellow mealworm (*T. molitor*) and of the black soldier fly (*H. illucens*). The concentrations of each metal were chosen to be 0.5, 1, and 2 times the maximum EC limits for complete animal feed. As soon as the first pupae developed, the feeding period was stopped, and the larval metal content was analyzed directly after harvest without any fasting period. No effects on the total larval weight, development time and survival rate were observed in any of the feeding groups for both insect larvae. To separate the metal fraction retained in the gut of the larvae from the metal fraction transferred to the insect tissue, in additional feeding groups at the highest metal dose larvae of both species were reared on uncontaminated feed for two days prior to analysis. Differences in the accumulation behavior were evident as larvae of the yellow mealworm substantially accumulated arsenic, whereas larvae of black soldier fly preferentially accumulate lead and especially cadmium. These results are consistent with the findings of Diener et al. [92]. For black soldier fly larvae, the arsenic concentrations remained below the maximum EC limit for animal feed, even when reared on two times the limit for arsenic in the feed and analyzed directly after harvest without being reared on uncontaminated feed. In contrast, for the yellow mealworm arsenic concentrations in the larvae were higher than in the residual material indicating that arsenic was retained in the body. When reared on feed at 2 mg/kg arsenic, the maximum EC level of arsenic in complete feed, the concentration in the larvae exceeded this limit already. Larvae of the feeding group with two times the EC limit in complete feed still exhibited a four times higher concentration of arsenic than the EC maximum level allowed in complete feed even after two days rearing on a clean substrate. Bioaccumulation factors of lead in the case of the black soldier fly larvae were closely above 1, thus, very similar to the concentration in the feed. In contrast, lead concentrations in the residual materials of yellow mealworm larvae were up to sixty times higher than the concentration detected in the larvae pointing to efficient elimination. In all feeding groups of the black soldier fly larvae, the cadmium concentrations were above 0.5 mg/kg, the maximum EC limit in complete feed, and always higher than in the residuals. The lower cadmium concentration found in the larvae of the yellow mealworm compared to the black soldier fly was related to the fact that the calcium content in black soldier fly is extraordinarily high enabling much more pronounced accumulation of cadmium than found in other dipterans [105]. Based on these findings, the authors addressed the need to re-establish the maximum limits of the tested metals in complete feed for each insect species to ensure safe use of these insects as feed materials.

Opposed to results by Diener et al. [92], van der Fels-Klerx et al. [93] and Purschke et al. [58], the presence of cadmium and chromium in the feed led to retarded larval development but no detrimental effects on survival rate of black soldier fly larvae (*H. illucens*) according to the study of Gao et al. [94]. At a lifelong supply of wheat bran spiked with chromium at 300 mg/kg feed and cadmium at 4.5 mg/kg both cadmium and chromium could be quantified after a 12 h fasting period in 12-day old larvae, in prepupae as well as in pupae with decreasing concentration in later development stages. While cadmium accumulated in the larval and prepupal stage indicated by higher concentrations in the body than in the initial feed in accordance with results by Diener et al. [92] and van der Fels-Klerx et al. [93], chromium concentrations in the body of the larvae and the prepupae were below that in the feed. In a metal analysis of one individual representative of the three different life stages considered, most of the cadmium and chromium was detected in the body of the larva and prepupa and not in the integument yet, with lower cadmium and chromium in the pupa body than in its puparium. However, as these data are based on only one individual organism of each development stage, it should be interpreted with care. Moreover, metal analysis of the larvae excretion should have been included in the study to better assess the fate of ingested heavy metals during larval development. Besides contamination by mycotoxins and pesticides, Purschke et al. [58] also investigated the bioaccumulation of several heavy metals in larvae of the black soldier fly (*H. illucens*). The larvae were reared for a period of 10 days on substrates spiked at metal concentrations commonly found in animal feed: Cadmium at a concentration of 1.5 mg/kg, lead at 15 mg/kg, mercury of 0.1 mg/kg, chromium at 15 mg/kg, nickel at 15 mg/kg and arsenic at 3 mg/kg. Survival rate did not show huge differences between the metals tested, while the growth performance of the larvae was inhibited noticeably which is consistent with reports from Diener et al. [92] and van der Fels-Klerx et al. [93]. In this study, larvae were analyzed directly after harvest without fasting or rearing on uncontaminated feed prior to analysis.

Evaluation of the bioaccumulation factors (concentration in the organism divided by the concentration in the substrate in analogy to [92]), revealed significant accumulation of lead and cadmium with bioaccumulation factors of two and nine, respectively. This resembles bioconcentration factors reported by other groups [93,106]. Approximately four-times lower concentrations of nickel and chromium were detected in the larvae compared with the feeding substrate, but slightly higher concentrations were found in the residual material pointing to efficient elimination. Neither could any accumulation be observed for arsenic with nearly constant concentrations in feed, larvae and residual substrate. Similarly, mercury did not accumulate in the larvae as slightly higher concentration the residual material was yielded than in the feed confirming the elimination of mercury by the larvae. Given the results of the study the authors concluded that during industrial production of black soldier fly larvae for the use as feed strict monitoring of heavy metal concentration, especially cadmium and lead, was required in the rearing substrate as well as in the larvae to ensure safe use and an economic process.

Likewise, with the focus of potential use in animal feed, Biancarosa et al. [95] evaluated the uptake of heavy metals and arsenic from a naturally contaminated seaweed using larvae of the black soldier fly (*H. illucens*). The metal contents were gradually increased by a stepwise replacement of the plant-based rearing substrate with the naturally contaminated seaweed ending at a maximum dose of 0.34 mg/kg cadmium, 0.25 mg/kg lead, 0.021 mg/kg mercury, and 36 mg/kg total arsenic of which only 0.09 mg/kg was inorganic arsenic. After eight days of rearing on the substrates, the larvae were analyzed without any further treatment. Replacing the control medium by more than 50% of contaminated seaweed had negative effects on larval growth performance and survival rates. However, the effect of uncontaminated seaweed on the biological performance of the larvae was not evaluated. Lead and mercury were detected in the larvae at concentrations which were linearly increasing with increasing dose levels and resembled the levels of the respective feeding substrate suggesting that uptake of lead and mercury was not strongly regulated.

The cadmium uptake as a function of cadmium dose in the feed followed a sigmoidal curve with a plateau at approximately 2 mg/kg. The uptake of arsenic increased linearly with the concentration in the feed. The majority of total arsenic is present in seaweed in the form of arseno-sugars of nearly unknown toxicity but may also contain inorganic arsenic [107]. As in the larvae also only <1% of total arsenic was present as inorganic form, it was assumed that larvae are not capable to convert organic forms of arsenic into inorganic arsenic. With respect to the maximum EC limits for undesirable substances such as heavy metals and arsenic in feed and feed materials, the allowed limits were already exceeded for cadmium (0.5 mg/kg) and arsenic (2 mg/kg) when 20% of the rearing materials was replaced by the naturally contaminated seaweed. The same group could show very recently, that replacing a fish meal with an insect meal produced from larvae of *H. illucens* that fed on media containing 60% seaweed lowered the accumulation of arsenic in the fillet of *Atlantic salmon* [108].

### 3.3. Rearing on Side Stream Substrates

Insects are also attracting attention because some of them can convert organic waste streams into valuable biomass under excretion of a compost-like residue similar to immature compost [109,110]. For example, black soldier fly larvae naturally feed on substrates of low or zero economic value such as animal manures, human excreta, fruit and vegetable wastes, as well as carrion [111,112]. Several literature data demonstrated that a variety of low-value substrates can be used as rearing substrate for insect larvae: Organic wastes comprising different compositions of fruits, legumes, and cereals could be used to grow larvae of *T. molitor* for the use as chicken feed [113]. Substrates composed of industrial organic by-products from beer brewing, bread and cookie backing, potato processing and bioethanol production could be used successfully to grow three different edible mealworm species [114]. Similarly, vegetable and fruit wastes, winery and brewery by-products were suitable as rearing substrates for black soldier fly larvae [115]. However, both studies focused on the growth performance and nutritional composition of the larvae without analysis of potential contaminants. Suitable protein quality was yielded by rearing larvae of the black soldier fly on distiller´s grains and dried sugar beet pulp, however, accumulation of the heavy metals cadmium and lead occurred and should be monitored [106]. Even sewage-sludge [116,117], animal manures [118,119] were used as a substrate for growing black soldier fly larvae. The use of black soldier fly larvae in terms of waste treatment technology has been recently reviewed by Gold et al. [120].

### 3.4. Conclusion on Accumulation Potential of Metal Contamination

The reported data revealed that essential metals could be efficiently regulated in insect larvae after ingestion, whereas regulation capacity for non-essential metals was much lower, leading to accumulation in the insects and adverse effects on survivability and growth performance. However, the accumulation behavior follows different patterns depending on insect species and insect developmental stage, as summarized in Table 6.

Accumulation of cadmium was consistently reported in larvae and prepupae of the black soldier fly larvae as indicated by a bioconcentration factor above one, while in adults no cadmium accumulation was observed. Larvae of the yellow mealworm, however, accumulated arsenic to a higher extent than cadmium. It could be shown that rearing substrates, that comply with the existing EC limit for cadmium in complete animal feed, were not sufficient to guarantee later safe use of insect larvae as animal feed or for human consumption [93]. Based on these findings, species-specific limitations regarding (heavy) metals in feed (and food) might be required to avoid the accumulation of heavy metals in the larvae.

## 4. Contaminants Identified in Commercially Available Insects and Insect-Based Products Used as Animal Feed or for Human Consumption

Charlton et al. [121] were exploring the chemical safety of four different species of fly larvae as a source of protein for animal feed. Larvae were sampled from institutes in UK, Mali, and China representing key production settings for insect larvae in Europe, Africa, and Asia. Regionally different production techniques from small scale production in Africa to large scale production in China were also covered by the study. In a broad screening program, only seven contaminants were found out of the considered 1140 contaminants potentially coming from veterinary drugs, pesticides, heavy metals, dioxins and polychlorinated biphenyls, polyaromatic hydrocarbons, and mycotoxins. Among the detected contaminants (4,4’-dinitrocarbanilide, chlorpyrifos, cadmium, Beauvericin, Enniatin A, Enniatin A1, piperonyl butoxide) only cadmium was above the maximum EC limit in animal feed of 0.5 mg/kg (three of nine samples analyzed). This finding confirms the cadmium accumulation shown in feeding studies under controlled lab conditions [58,92,93,94,95]. Due to (a) the low levels detected in the insects and (b) the lack of an EC regulation for the detected mycotoxins in feed, according to the authors the mycotoxins Beauvericin, Enniatin A, Enniatin A1 were not supposed to be a safety risk when using the insect larvae as animal feedstock. In a commercial sample of partially defatted insect meal obtained from *H. illucens* larvae, the metal content found was ten times below the maximum EC limit for arsenic, cadmium, lead, and mercury [122].

So far, only few data have been published on contaminants identified in commercially available insects and insect-based products for human consumption. In a study of Vandeweyer et al. [123] mycotoxin-forming fungi from *Aspergillus* spp. and *Penicillium* spp. could be found in the substrate and the insect body of *Gryllodes sigillatus* (Orthoptera: Gryllidae) crickets which were industrially reared for human consumption. In the substrate, isolates were identified as *Aspergillus, Candida, Lichtheimia, Penicillium*, and *Trichoderma* species, while the isolates from the crickets were identified as *Aspergillus, Candida*, *Kodamaea, Lichtheimia, Tetrapisispora, Trichoderma*, and *Trichosporon* species [123].

In realistic samples of dried edible insects from markets of Zambia, total aflatoxin concentrations (including aflatoxins B1, G1, B2, and G2) were slightly above the local regulatory limits of 10 µg/kg (detected in moths and termites). These values increased to considerably higher levels after simulated poor storage under elevated temperature and humidity [124]. Regarding metal contamination, the analysis of, e.g., nickel, lead, cadmium, zinc, and copper in African edible insects did not discover toxic levels of each metal [125]. Poma et al. [126] focused on commercially available edible insects and insect-based food products that were purchased between November 2015 and February 2016 from different European contributors. The concentration of organic contaminants was generally low and even lower than detected in animal products. Likewise, heavy metals were present in very low concentrations. The values of arsenic, lead and cadmium were below the concentrations found in edible grasshopper in Korea [127]. Copper and zinc levels were in the range typically occurring in meat and fish. These results confirm that edible insects comprise a valuable source of these essential micronutrients [128]. Based on these analytical results, Poma et al. [126] concluded that farmed insects could potentially accumulate chemicals. However, as the levels of contaminants in the tested samples were very low, no additional hazard to humans by consumption of edible insects and insect-based products compared to animal products was assumed.

## 5. Conclusions and Outlook

This review article was supposed to contribute to the feed and food safety assessment of insects for the use in animal feed and for human consumption by summarizing relevant legal aspects and reviewing the available literature data with a focus on mycotoxins and heavy metals which might be encountered during commercial insect farming. While the insect species itself intended to be used for feed or food in most cases do not pose an imminent risk to animals or humans, the major risk was identified to originate from the rearing substrate used in the insect farming. Substrates contaminated with mycotoxins or heavy metals may cause adverse effects on survivability and growth performance of the insects. Accumulation of mycotoxins has not been observed so far in any insect species of interest. However, a considerable portion of mycotoxin ingested could not be recovered in the respective studies. Furthermore, literature data on metabolism pathways of mycotoxins in insects is still very limited and further research on that aspect is urgently needed. Contrary, metal accumulation does occur to a varying extent dependent on metal type, insect species, and developmental stage. According to literature data, the black soldier fly is capable to significantly accumulate cadmium, whereas the yellow mealworm rather accumulates arsenic in the larval body. Hence, extrapolation of experimental data generated with one insect species to other insect species is difficult to estimate due to the different metabolic and physiological characteristics between insect species. The reported data emphasize the importance to assess potential food safety hazards in a case-by-case approach, as a possible accumulation of contaminants strongly depends on the insect species and the developmental stage of the insects. With respect to the rearing substrate, regular monitoring of contaminants is an essential part of feed and food security. Considering that some insects are capable to convert organic waste into valuable biomass, the ban of using waste streams in animal feed according to EU regulation might be revised in future with respect to the special case of insect farming for feed and food. Moreover, the current EU limits for contaminants such as heavy metals in animal feed, might need to be adapted according to the species-specific accumulation behavior. Besides the evaluation of mycotoxins and heavy metals, a comprehensive safety assessment of microbial hazards, chemical hazards (considering contamination with pesticides and veterinary medicines), as well as the allergenic potential of edible insects and derived products, needs to be conducted for each insect species. An exemplary species-specific risk profile has recently been published for the house cricket (*Acheta domestica* (Orthoptera: Gryllidae)) [3]. Biological and chemical hazards were ranked by the authors in the category “low”, “medium”, and “high” according to risk ranking framework on biological hazards by EFSA [129]: Medium hazards identified were high total aerobic bacterial counts, survival of spore-forming bacteria following thermal treatment, allergenicity of insect and insect-derived products and the accumulation of heavy metals, especially cadmium. In contrast, parasites, fungi, viruses, prions, antimicrobial resistance, and the presence of toxins such as mycotoxins were ranked as low risk.

## Figures and Tables

**Table 1:** Selection of insect species with the potential to be used as food or feed including information on the reported application in commercial farming within and outside Europe [6].

Insect Order	Insect Species	Life-Stage at Harvest	Farmed for
Diptera	black soldier fly *(Hermetia illucens)*	larvae, prepupae, pupae	feed
Diptera	common housefly *(Musca domestica)*	larvae	feed
Diptera	yellow mealworm *(Tenebrio molitor)*	larvae	feed, food
Coleoptera	superworm *(Zophobas atratus)*	larvae	feed, food
Coleoptera	giant mealworm *(Zophobas morio)*	larvae	feed
Coleoptera	lesser mealworm *(Alphitobius diaperinus)*	larvae	feed, food
Lepidoptera	silkworm *(Bombyx mori)*	prepupae, pupae	feed, food
Orthotpera	house cricket *(Acheta domesticus)*	adult	feed, food
Orthoptera	banded cricket *(Gryllodus sigillatus)*	adult	feed
Orthoptera	African migratory locust *(Locusta migratoria migratorioides)*	adult	feed, food
Orthoptera	American grasshopper *(Schistocerca Americana)*	adult	feed, food

**Table 2:** Overview of regulatory framework regarding food and feed in the European Union.

Regulation	Key Aspect of European Regulation/Directive
315/93/EEC	Food containing a contaminant in an amount which is unacceptable from the public health viewpoint and in particular at a toxicological level shall not be placed on the market, contaminant levels shall be kept as low as can reasonably be achieved (ALARA principle) by following good practice.
EC 178/2002	Food shall not be placed on the market if it is unsafe, i.e., injurious to health, unfit for human consumption.
EC 1881/2006	Maximum levels for certain contaminants (nitrate, mycotoxins, citrinin, ergot sclerotia and ergot alkaloids, metals, 3-monochloropropanediol (3-MCPD) and glycidyl fatty acid esters, dioxins and PCBs, Polycyclic aromatic hydrocarbons, melamine, and its structural analogs and inherent plant toxins) in foodstuffs.
EU 2015/2283	Novel foods regulation, among others whole insects and their parts constitute novel foods.
EC 999/2001	Rules for the prevention, control, and eradication of certain transmissible spongiform encephalopathies, prohibits processed animal protein as feed for farmed animals.
EU 2017/893	Amendment of Annexes I and IV to EC 999/2001 permits processed animal protein derived from farmed insects as feed material for aquaculture animals.
2002/32/EC	Maximum limits of undesirable substances (e.g., metals, mycotoxins, dioxins) in animal feed.
2006/576/EC	Guidance values for deoxynivalenol, zearalenone, ochratoxin A, T-2 and HT-2 and fumonisins in products intended for animal feeding.
EC 1069/2009	Health rules as regards animal by-products and derived products not intended for human consumption, defines farmed animal as any animal that is kept, fattened or bred by humans and used for the production of food, wool, fur, feathers, hides, and skins or any other product obtained from animals or for other farming purposes.
EC 767/2009	Conditions for the placing on the market and the use of feed, in order to ensure a high level of feed safety and thus a high level of protection of public health, prohibits the use of various waste materials as animal feed.

**Table 3:** EU maximum limits [21] for cadmium, lead, arsenic, mercury, and aflatoxin B1 and guidance values [22] for deoxynivalenol, zearalenone, fumonisins B1 and B2, and ochratoxin A in selected feed materials and complete feed for farm animals in mg/kg relative to a moisture content of 12%.

Contaminant	Products Intended for Animal Feed	Maximum Content [mg/kg] [21]	Guidance Value [mg/kg] [22]
Cd	A B	2 0.5	
Pb	A B	10 5	
As	A of animal origin B	2 2	
Hg	A B	0.1 0.1	
Aflatoxin B1	A B C for dairy animals and young animals	0.02 0.01 0.005	
Deoxynivalenol	D E F for pigs		8 12 0.9
Zearalenone	D E F for piglets and gilts		2 3 0.1
Fumonisin B1 + B2	E F for pigs		60 5
Ochratoxin A	D F for pigs		0.25 0.05

A: Feed materials, B: Complete feed, C: Compound feed, D: Cereals and cereal products, E: Maize by-products, F: Complementary and complete feed.

**Table 4:** Overview of feeding studies with mycotoxins (controlled conditions).

Larvae Species	Rearing Substrates	Duration of Feeding Period	Analytes	Treatment Prior to Analysis	Reference
*Alphitobius diaperinus, Tenebrio molitor, Zophobas atratus*	*Tenebrio molitor* and *Zophobas atratus*: Wheat bran spiked with Zearalenone, Ochratoxin A, T-2 toxin at 500 µg/kg *Alphitobius diaperinus*: Diet containing maize, wheat, soy, limestone, palm-, sunflower- and soybean oil, spiked with Zearalenone, Ochratoxin A, T-2 toxin at 500 µg/kg	*Alphitobius diaperinus*: 15 days *Tenebrio molitor*: 28 days *Zophobas atratus*: 40 days (depending on species-specific developmental time)	zearalenone (ZEN) ochratoxin A (OTA) T-2 toxin	analyzed directly vs. 24 h/48 h/72 h fasting	[54]
*Tenebrio molitor*	wheat flour Naturally contaminated with mycotoxins at levels of 4.9 mg/kg deoxynivalenol (DON), 86 µg/kg 15-ADON, 300 µg/kg DON-3-glucoside) spiked with 8 mg/kg DON	14 days	deoxynivalenol (DON) DON-3-glycoside (DON-3G) 15-acetyl-DON (15-ADON)	analyzed directly vs. 24 h fasting	[55]
*Tenebrio molitor*	Naturally contaminated grain at levels of 0.2 ppm, 2 ppm, 10 ppm, and 12 ppm DON	32.8 ± 3.2 days (until 2 pupae were observed)	deoxynivalenol (DON) 3-acetyl-DON (3-ADON) Nivalenol (NIV)	24 h fasting	[56,57]
*Hermetia illucens*	Corn semolina-based substrate spiked with 4.6 mg/kg DON, 88 µg/kg AfB1, 17 µg/kg AfB2, 46 µg/kg AfG2, 260 µg/kg OTA, 860 µg/kg ZEN	10 days	aflatoxin B1 (AfB1) aflatoxin B2 (AfB2) aflatoxin G2 (AfG2) DON OTA ZEN	analyzed directly without fasting	[58]
*Hermetia illucens, Tenebrio molitor*	Poultry feed spiked with AfB1 at levels of 0.01, 0.025, 0.05, 0.10, 0.25, and 0.5 mg/kg dry feed	*Hermetia illucens*: 10 days *Tenebrio molitor:* Until first pupa was observed	AfB1 aflatoxin M1 (AfM1)	analyzed directly (T. molitor) vs. 2 days on non-contaminated feed	[59]
*Alphitobius diaperinus, Hermetia illucens*	Commercial wheat-based rearing substrate spiked with AfB1, DON, OTA and ZEN in concentrations of 1, 10, and 25 times the maximum EC limits or guidance values for specific complete feed spiked with mixtures of mycotoxins (with an average of 8- to 20-fold increase of the EU limits)	*Alphitobius diaperinus*: 14 days *Hermetia illucens*: 10 days (depending on species-specific developmental time)	AfB1 aflatoxicol aflatoxin P1 (AfP1) aflatoxin Q1 (AfQ1) AfM1 DON 3-acetyl-DON (3-ADON) 15-ADON DON-3G ZEN α-zearalenol (α-ZEL) β-zearalenol (β-ZEL)	2 days on non-contaminated feed	[60]
*Tenebrio molitor*	Wheat flour spiked with toxins at levels of approx. 500 µg/kg ZEN and approx. 2000 µg/kg ZEN blended with artificially contaminated wheat flour at levels of approx. 500 µg/kg ZEN and approx. 2000 µg/kg ZEN blended with naturally contaminated wheat flour at levels of approx. 600 µg/kg ZEN and approx. 900 µg/kg ZEN	4 weeks (short-term trial) and 8 weeks (long-term trial)	ZEN ZEN-14-*O*-glucuronide ZEN-14-sulfate (ZEN14Sulf) ZEN-14-*O*-glucoside ZEN-16-*O*-glucoside hydrolyzed ZEN decarboxylated hydrolyzed ZEN α-ZELα-ZEL-14-*O*-glucuronide α-ZEL-sulfate (ZELSulf) β-ZEL β-ZEL-14-*O*-glucuronide α-/β-zearalanol (ZAL) Zearalanone (ZAN) DON	24 h fasting	[61]

**Table 5:** Overview of feeding studies with different metals under controlled conditions.

Larvae Species	Rearing Substrate	Duration of Feeding Period	Analytes	Treatment Prior Analysis	Reference
*Tenebrio molitor*	Experiment 1: Single dose of ^109^Cd on potato Experiment 2: Bread baked with Cd at 0.1, 5, 15, 30 mg/kg	Experiment 1: 1 day Experiment 2: Until 2nd or 3rd instar larvae reached final larval stage	Cd	experiment 2: 24 h fasting	[91]
*Hermetia illucens*	Chicken feed spiked with Cd at 2 mg/kg, 10 mg/kg, 50 mg/kg Pb at 5 mg/kg, 25 mg/kg, 125 mg/kg Zn at 100 mg/kg, 500 mg/kg, 2000 mg/kg	Entire lifetime from larvae to adult stage	Cd Pb Zn	analyzed directly after harvest	[92]
*Tenebrio molitor,* *Hermetia illucens*	Feed spiked with Cd at 0.25 mg/kg, 0.5 mg/kg, 1 mg/kg Pb at 2.5 mg/kg, 5 mg/kg, 10 mg/kg As at 1 mg/kg, 2 mg/kg, 4 mg/kg(concentrations 0.5, 1, 2 times the maximum EC limit in complete feed)	Until first pupa was observed	Cd Pb As	analyzed directly after harvest vs. 2 days on uncontaminated feed for highest dose groups	[93]
*Hermetia illucens*	Wheat bran spiked with 4.5 mg/kg Cd 300 mg/kg Cr	As soon as >40% reached prepupal stage (indicated by darkening of the integument)	Cd Cr	12 h fasting	[94]
*Hermetia illucens*	Corn semolina-based substrate spiked with 1 mg/kg Cd, 10 mg/kg Pb, 0.1 mg/kg Hg, 10 mg/kg Cr, 10 mg/kg Ni, 2 mg/kg As	10 days	Cd Pb Hg Cr Ni As	analyzed directly after harvest	[59]
*Hermetia illucens*	Plant-based growth medium replaced gradually in 10% steps by seaweed containing 0.34 mg/kg Cd, 0.25 mg/kg Pb, 0.021 mg/kg Hg, 36 mg/kg total As (0.09 mg/kg inorganic As)	8 days (until control group reached harvest size)	Cd Pb Hg As	analyzed directly after harvest	[95]

**Table 6:** Overview of literature data on bioaccumulation factors (BAFs) for cadmium and arsenic in *Tenebrio molitor* and *Hermetia illucens*.

Species	Mean Bioaccumulation Factors	Gut Clean Prior Analysis	Reference
*Tenebrio molitor*	0.8–1.05 (larvae, Cd) ^1^ 0.3–0.38 (adult, Cd) ^1^	yes	[91]
*Hermetia illucens*	2.46–2.79 (larvae, Cd) 0.86–1.41 (larval exuviae, Cd) 2.32–2.94 (prepupae, Cd) 0.13–0.21 (adults, Cd)	no	[92]
*Tenebrio molitor*	0.65 ± 0.037–0.71 ± 0.039 (larvae, Cd) 1.4 ± 0.045–2.6 ± 0.23 (larvae, As)	no	[93]
*Hermetia illucens*	9.5 ± 3.6–6.1 ± 1.9 (larvae, Cd) 0.49 ± 0.10–0.58 ± 0.12 (larvae, As)	no	[93]
*Hermetia illucens*	4.635 (larvae, Cd) 4.198 (prepupae, Cd) 0.507 (pupae, Cd)	no	[94]
*Hermetia illucens*	9.1 ± 1.4 (larvae, Cd)	no	[58]
*Hermetia illucens*	4.5–8.33 (larvae, Cd) ^1^	no	[95]

^1^ BAF values derived by dividing the Cd concentration measured in the insect by the Cd concentration provided in the feed.
